# Tuberculosis Epidemiology in Islands: Insularity, Hosts and Trade

**DOI:** 10.1371/journal.pone.0071074

**Published:** 2013-07-29

**Authors:** Pelayo Acevedo, Beatriz Romero, Joaquin Vicente, Santo Caracappa, Paola Galluzzo, Sandra Marineo, Domenico Vicari, Alessandra Torina, Carmen Casal, Jose de la Fuente, Christian Gortazar

**Affiliations:** 1 Centre de Recerca en Sanitat Animal, Campus de la Universitat Autònoma de Barcelona-IRTA, Bellaterra (Cerdanyola del Vallés), Spain; 2 SaBio-IREC (University of Castilla-La Mancha-Consejo Superior de Investigaciones Científicas-Junta de Comunidades de Castilla - La Mancha), Ciudad Real, Spain; 3 Centro de Investigação em Biodiversidade e Recursos Genéticos/InBio Laboratório Associado, Universidade do Porto, Vairão, Portugal; 4 Centro de Vigilancia Sanitaria Veterinaria, Veterinary School, Complutense University of Madrid, Madrid, Spain; 5 Istituto Zooprofilattico Sperimentale della Sicilia, Palermo, Italy; 6 Department of Veterinary Pathobiology, Center for Veterinary Health Sciences, Oklahoma State University, Stillwater, Oklahoma, United States of America; Centers for Disease Control and Prevention, United States of America

## Abstract

Because of their relative simplicity and the barriers to gene flow, islands are ideal systems to study the distribution of biodiversity. However, the knowledge that can be extracted from this peculiar ecosystem regarding epidemiology of economically relevant diseases has not been widely addressed. We used information available in the scientific literature for 10 old world islands or archipelagos and original data on Sicily to gain new insights into the epidemiology of the *Mycobacterium tuberculosis* complex (MTC). We explored three nonexclusive working hypotheses on the processes modulating bovine tuberculosis (bTB) herd prevalence in cattle and MTC strain diversity: insularity, hosts and trade. Results suggest that bTB herd prevalence was positively correlated with island size, the presence of wild hosts, and the number of imported cattle, but neither with isolation nor with cattle density. MTC strain diversity was positively related with cattle bTB prevalence, presence of wild hosts and the number of imported cattle, but not with island size, isolation, and cattle density. The three most common spoligotype patterns coincided between Sicily and mainland Italy. However in Sicily, these common patterns showed a clearer dominance than on the Italian mainland, and seven of 19 patterns (37%) found in Sicily had not been reported from continental Italy. Strain patterns were not spatially clustered in Sicily. We were able to infer several aspects of MTC epidemiology and control in islands and thus in fragmented host and pathogen populations. Our results point out the relevance of the intensity of the cattle commercial networks in the epidemiology of MTC, and suggest that eradication will prove more difficult with increasing size of the island and its environmental complexity, mainly in terms of the diversity of suitable domestic and wild MTC hosts.

## Introduction

Island ecology is different from that of mainland systems. In the seminal work of MacArthur and Wilson [1] the main ecological peculiarities of islands were compiled and put in a biogeographical context. This was the starting point for the development of island biogeography [2]. Recently, Losos and Ricklefs [3] reinvigorated the original theory and described how the field has extended in the last decades. Now, even if there is taxonomic bias in favour of vertebrates in island biogeographical studies as compared to scientific publications as a whole [4], many recent studies on this framework were focused on other groups of living beings [5]–[7].

Communities of parasites–in a broad sense [8]–were also studied in the analytical framework of island biogeography. Most of these studies were focused on the co-evolution in the parasite-host/s systems [9], parasite distribution patterns and assemblages [10]–[12], and on the parasite traits that prevail in island ecosystems [13]. Other authors considered the individuals/populations of a given host species as–biological–islands [14], and then used the principles of island biogeography to study, for example, the transmission pathways among individuals/populations and the persistence in each one [15]. Regarding infectious diseases that can be transmitted from animals to humans–i.e. zoonotic diseases–Reperant [16] described the advantages of the theoretical framework of island theory, in the context of “biological islands”, to study the emergence of zoonotic diseases. Several relevant points can be raised from her review in order to approximate biogeographical principles and epidemiological studies. Indeed, islands are for instance a good target for control/eradication programs [17]. However, to our best knowledge, the epidemiology of economically relevant diseases on geographic islands and the new insights that can be extracted from this peculiar ecosystem has not been addressed in detail.

Here, we studied the *Mycobacterium tuberculosis* complex (MTC) epidemiology in the old world islands and archipelagos (hereafter islands). MTC is composed of a diversity of bacterial organisms belonging to several species including the typically human pathogen *M. tuberculosis*, and mainly animal ones such as *M. bovis* and *M. caprae*. The MTC is a highly clonal group of strains [18], and several clonal groups have so far been described in Europe [19] and elsewhere. Members of the MTC are characterized by low (though variable) host specificity, and particularly *M. bovis* is able to infect multiple host species [20]. This ability of *M. bovis* to infect a broad range of host species contributes to the view that the control of tuberculosis in cattle (mainly caused by *M. bovis*) will not be achieved if wildlife reservoirs of infection are present [21]. Bovine tuberculosis (bTB) is a zoonotic infectious disease with important consequences for livestock trade. In developed countries, the main concerns regarding bTB are the severe economic losses to the cattle industry and governments, arising from control costs and movement restrictions [22]. Moreover, MTC members change their genetic characteristics in time, mainly through small genome deletions that enable the use of molecular typing techniques in epidemiology [23]–[24]. Studies carried out on different spatial scales suggested that the different *M. bovis* typing patterns are not randomly distributed in space and then by studying their spatial-temporal patterns several inferences on this complex epidemiological system were extracted [24]–[26]. These characteristics make MTC an interesting subject to be studied from a biogeographical perspective.

Regarding MTC in the old world, islands can largely be considered as epidemiological units, where the prevalence and MTC type diversity will largely depend on natural factors such as island size and isolation, and, eventually, local adaptation and diversification, but also on other factors such as habitat, host diversity and density, and the human influence through cattle trade and test and slaughter policy [10], [13], [27]. Islands have occasionally been used to study *M. tuberculosis* epidemiology in humans [28]–[29], and a few studies have addressed the epidemiology of *M. bovis* on islands [28], [30]–[31]. However, the information that can be extracted from insular ecosystems regarding epidemiology of cattle bTB has not been addressed in detail. Herein, we reviewed data on MTC in animal hosts from 10 old world islands to gain new insights into MTC epidemiology. We explored three nonexclusive working hypotheses on the processes modulating bTB prevalence and MTC strain diversity in islands: insularity, hosts and trade.

## Materials and Methods

### Study areas: bTB prevalence and MTC type diversity

**Figure 1:**
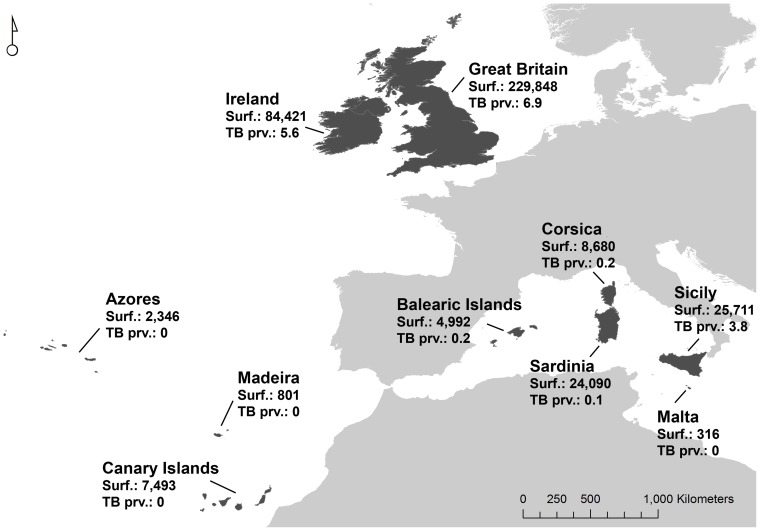
Islands and archipelagos considered in the study. Location, surface and cattle herd tuberculosis prevalence (see Table 1 for data sources) in the study islands and archipelagos are shown.

We used information available in the scientific literature for 10 old world islands or archipelagos (Fig. 1). The Mediterranean islands (Balearic Islands in Spain, Corsica in France, Sardinia and Sicily in Italy, and Malta) are known since antiquity and cattle and goats have probably been present for millennia. The same holds for the two large Atlantic islands, Great Britain and Ireland. All above mentioned islands also have in common a certain proximity to their respective mainland. By contrast, the three Atlantic archipelagos Azores and Madeira (in Portugal), and Canary Islands (in Spain) are further away from their historical mainland and were colonized more recently (1427 for Azores, 1418 for Madeira, 1402 for The Canaries). Table 1 summarizes the information of epidemiological relevance available for these islands. Among other variables, the cattle herd bTB prevalence and the number of different MTC strains in relation to the number of isolates, were recorded.

**Table 1:** Relevant epidemiological characteristics of the ten islands or archipelagos included in the study (see also Fig. 1).

Island or archipelago	Number of MTC strains	References
Great Britain	34 strains in 9839 isolates	http://www.mbovis.org/spoligodatabase/GBmetadata/frequency%20spoligo%20GB.html. Accessed 2013 Jul 3.
Ireland	29 strains in 503 isolates	[67]–[68] http://www.dardni.gov.uk/index/statistics/animal-disease-statistics/statistics-tuberculosis.htm. Accessed 2013 Jul 3.
Sicily	19 strains in 145 isolates	http://www.salute.gov.it/relazioneAnnuale2010/paginaInternaSottomenuRelazioneAnnuale2010.jsp?id=482&lingua=italiano&menu=cap2&sottomenu=162. Accessed 2013 Jul 3.
Sardinia	No information available	http://www.salute.gov.it/relazioneAnnuale2010/paginaInternaSottomenuRelazioneAnnuale2010.jsp?id=482&lingua=italiano&menu=cap2&sottomenu=162. Accessed 2013 Jul 3.
Corsica	3–4 strains in <10 isolates	[62], [69]
Canary Islands	4 strains in 55 isolates	[70]; http://rasve.magrama.es. Accessed 2013 Jul 3.
Balearic Islands	3 strains in 8 isolates	[70]; http://rasve.magrama.es. Accessed 2013 Jul 3.
Azores	4 strains in 20 isolates	[71]; but outbreak in 2007 see Matos et al. [30]
Madeira	0 types, TB free	[71]
Malta	0 strains, TB free	http://www.efsa.europa.eu/en/scdocs/doc/zoon_report_2005_Malta_en.pdf. Accessed 2013 Jul 3.

The number of strains is based on spoligotyping.

Since both MTC type richness and island size could be correlated with sampling effort [32], we did not consider specific richness (*S*) as the sum of MTC types detected. We estimated Margalef's diversity index [33], since it accounts for sampling size (*N*) in the following way: 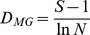
_._

Even when other diversity indices are more robust than Margalef's one [34], we used this since the information requested for others, e.g., the types isolated in each sample, was not available in this study. Here *S* and *N* were obtained from literature (see Table 1).

### Insularity, host density and trade

Three nonexclusive working hypotheses on the processes modulating bTB prevalence and MTC type diversity in islands were comparatively assessed in this study: insularity, i.e., island size and isolation (hypothesis 1), host density, i.e., density of cattle and presence of potential wild host species (hypothesis 2), and trade, i.e., number of cattle imported (hypothesis 3). According to the theory of island biogeography [1], bTB prevalence and MTC strain diversity in islands could be modulated by island size and isolation (measured as the minimum distance to mainland). Host density is a major source determining parasite burdens [35]. Thus, density of cattle and presence of potential wild host species (deer, badgers [*Meles meles*], and wild boar [*Sus scrofa*]; see [36]) could be related to bTB prevalence and MTC diversity in islands. The census of cattle in each island was obtained from the [national] institutes of statistics (Table 2). Total numbers were converted into cattle densities (heads/km^2^) by considering the surface of the island (Fig. 1). Finally, since movements of infected animals are a critical factor in the spread of livestock diseases [37], their role in the epidemiology of bTB is expected to be relevant. We were looking for a unique data source to obtain all the required information in a fully standardized way, but to our best knowledge, data on cattle movements are only centralized at country level (not for smaller regions). Thus, data on the number of live cattle imported were extracted from [national] trade statistics websites (see Table 2). For some of the islands the information was referred to €. In these cases we transformed € into number of heads using unitary prices obtained from http://datacomex.comercio.es. Information was available for all islands with the exception of the Portuguese ones (Azores and Madeira). So, knowing the number of animals imported to Portugal in a year (http://datacomex.comercio.es. Accessed 2012 Feb 23), we estimated a number of animals imported for these islands by assuming this to be proportional to the number of heads in these islands in relation to the whole country. The notable differences among islands in relation to imported animals increase our confidence in that the ranking of islands, and therefore the results of the models, were not affected by our imprecise estimations for Azores and Madeira.

**Table 2:** Data used for modelling purposes, the year to which the data refers (between brackets) and the source of the information (see text for details).

Island or archipelago	Suitable maintenance hosts	Cattle density, heads per km^2^	Number of cattle imported in a year
Great Britain	cattle, badger, deer, wild boar	43.07 (2011) http://www.defra.gov.uk/statistics/foodfarm/landuselivestock. Accessed 2013 Jul 3.	79,219 (2011) https://www.uktradeinfo.com. Accessed 2013 Jul 3.
Ireland	cattle, badger, deer	79.55 (2009) http://www.cso.ie/en/statistics. Accessed 2013 Jul 3.	191,117 (2011) http://datacomex.comercio.es. Accessed 2013 Jul 3.
Sicily	cattle, goat, pig, fallow deer, wild boar	13.07 (2010) http://www.istat.it/it/archivio/32618. Accessed 2013 Jul 3.	102,804 (2011) Anagrafe Nazionale Zootecnica–Statistiche [restricted access]
Sardinia	cattle, goat, pig, wild boar, deer	10.43 (2010) http://www.istat.it/it/archivio/32618. Accessed 2013 Jul 3.	1169 (2011) Anagrafe Nazionale Zootecnica–Statistiche [restricted access]
Corsica	cattle, goat, pig, wild boar, deer	9.11 (2010) http://www.insee.fr/fr/themes. Accessed 2013 Jul 3.	86 (2011) Stéphanie Devaux–reponsible for tuberculosis in Camargue-Corse
Canary Islands	cattle, goat	2.07 (2009) http://www.ine.es/censoagrario/censoag.htm. Accessed 2013 Jul 3.	139 (2011) http://aduanas.camaras.org/. Accessed 2013 Jul 3.
Balearic Islands	cattle	6.59 (2009) http://www.ine.es/censoagrario/censoag.htm. Accessed 2013 Jul 3.	11 (2009) http://aduanas.camaras.org/. Accessed 2013 Jul 3.
Azores	cattle, goat	112.96 (2011) http://www.ine.pt/xportal. Accessed 2013 Jul 3.	2156 (2011) http://www.ine.pt/xportal [estimated]. Accessed 2013 Jul 3.
Madeira	cattle, goat	6.24 (2011) http://www.ine.pt/xportal. Accessed 2013 Jul 3.	37 (2011) http://www.ine.pt/xportal [estimated]. Accessed 2013 Jul 3.
Malta	cattle, goat	47.70 (2011) http://www.nso.gov.mt/site/page.aspx?pageid=30. Accessed 2013 Jul 3.	0 (2011) http://datacomex.comercio.es. Accessed 2013 Jul 3.

General linear models [38] were used to assess the capacity of the working hypotheses in explaining bTB prevalence and MTC type diversity (our response variables). For each of the response variables, independent models were fitted for insularity, host density and trade, and their performance was compared in terms of Akaike Information Criteria, corrected for small sampling size (AICc; [39]). Residuals of the models were examined and tested for normality using Kolmogorov-Smirnov test and for autocorrelation using the Moran's I in order to detect spatial structures [40]. We used Bonferroni-corrected significance levels.

### Case study Sicily: epidemiology of MTC strains

Sicily has a rich history with, in time sequence, Greek, Roman, Byzantine, Muslim, Norman, German (Hohenstaufen) and Spanish influence, until the final unification of Italy [41]. This historical complexity and the Island's relevance for trade made Sicily a perfect mixture vessel of MTC strains of different origin.

In Sicily, as in other Mediterranean areas, livestock represents one of the most important resources for the island economy. Extensive grazing methods represent an ancient, traditional practice for using poor lands. This sector involves more than 16,000 farms of cattle and 10,000 farms of sheep and goats (respectively 6% and 15% of the Italian national herds). In the period 1995–1999 more than 170 bTB outbreaks were notified although the pathogen prevalence and economical impact in the Sicilian livestock was still unknown [42]. According to the Italian Ministry of Health, in 2010 the proportions of infected cattle herds around the Sicilian provinces of Catania and Messina were 8.6% and 5.4%, respectively [43]. In addition to the “traditional” domestic MTC hosts cattle and goats, Sicily has large populations of free-roaming domestic pigs (Sicilian black pigs or Nebrodi black pigs), which are hypothesized to act as a reservoir of bTB in this ecological setting [43]. Moreover, native wild boar are also present on Sicily and could play a role in MTC epidemiology. Fallow deer (*Dama dama*), also suitable MTC hosts, were introduced from the Italian mainland in the late 20th Century and are maintained in two small nature reserves destined for scholar visits [44].

No animals were specifically sampled for this study. A total of 147 epidemiologically unrelated MTC isolates obtained from cattle (N = 145) and fallow deer (N = 2) were supplied by the Istituto Zooprofilattico Sperimentale (IZS). Animal samples were collected under the routine bTB testing and eradication scheme in Italy, that is coordinated by IZS in Sicily. This scheme in Italy is based on European Union (Council Directive 64/432/EEC, Annexes A, I and B) and Italian legislation (DM 592/95 and DLgs 196/99). In order to research for epidemiological backgrounds, typing methods were performed by the spoligotyping technique ([45]; N = 147). The nomenclature for the spoligotype patterns was obtained from the *M. bovis* Spoligotype Database website [46]. Results obtained in Sicilian cattle in terms of MTC strain community were compared with data available from the literature. In addition, we used SaTScan software (Martin Kulldorff, Harvard Medical School) to analyze the spatial structure in MTC strains and detect clusters of the strains on Sicily. We used a Bernoulli model considering as cases the three most prevalent strains (SB0120, SB0134 and SB0841) and the others (non SB0120, SB0134 and SB0841, respectively) as controls. Data was aggregated at farm level.

## Results

### Cattle bTB herd prevalence

In relation to hypothesis 1, cattle bTB herd prevalence correlated positively with island size (F_1,7_ = 17.540, p = 0.004) but not with isolation (F_1,7_ = 1.146, p = 0.320). That is, independently of isolation, larger islands had higher prevalence than small ones. This model attained a R^2^ = 0.728 and AICc = 47.61. Removing the non-significant variable, the same result was obtained for island size (F_1,8_ = 24.487, p = 0.001) and the performance of the final model was R^2^ = 0.723 and AICc = 43.13.

Assessment of hypothesis 2 showed that bTB prevalence was significantly related with the presence of wild hosts (F_1,7_ = 6.096, p = 0.043) but not with cattle density (F_1,7_ = 1.278, p = 0.296). Islands with presence of wild hosts had a higher prevalence than those without these potential hosts. This model attained a lower performance than the previous one (R^2^ = 0.361 and AICc = 56.15). Removing the non-significant term, wild hosts remained as significantly related to bTB prevalence (F_1,8_ = 5.606, p = 0.045) and the final model performance improved slightly (R^2^ = 0.339 and AICc = 51.83).

The bTB prevalence correlated with the number of imported animals (F_1,8_ = 22.640, p = 0.001); the higher the number of imported animals, the higher the bTB prevalence (hypothesis 3). The final model attained a R^2^ = 0.706 and AICc = 43.71.

The residuals of the three final models (with normal distribution in all cases) were not spatially structured according to Moran's I index. Finally, MTC type diversity was positively related with bTB prevalence (F_1,7_ = 32.951, p = 0.001, R^2^ = 0.800), that is, the higher the prevalence, the higher the diversity of MTC types.

### MTC strain diversity

In relation to hypothesis 1, the diversity of MTC strains was not statistically related with island size (F_1,6_ = 2.893, p = 0.140) nor isolation (F_1,6_ = 0.980, p = 0.360). The model attained a R^2^ = 0.343 and AICc = 45.53. Removing the isolation, island size was not significantly related with MTC diversity (F_1,7_ = 5.288, p = 0.055) and the performance of the final model was R^2^ = 0.349 and AICc = 39.69.

Assessment of hypothesis 2 showed that, similarly to the model for bTB prevalence, MTC diversity was significantly related with the presence of wild hosts (F_1,6_ = 13.306, p = 0.011) but not with cattle density (F_1,6_ = 1.404, p = 0.281). Islands with presence of wild hosts had a higher diversity of MTC strains than those without wild hosts. The model attained a higher performance than the previous one (R^2^ = 0.616 and AICc = 40.75). Removing the non-significant term, wild host presence remained as significantly related to MTC diversity (F_1,7_ = 12.704, p = 0.009) and the final model performance improved slightly (R^2^ = 0.594 and AICc = 35.45).

Finally, the MTC strain diversity was significantly correlated with the number of imported animals (F_1,7_ = 35.983, p = 0.001); the higher the number of imported animals, the higher the bTB prevalence (hypothesis 3). The final model attained a R^2^ = 0.814 and AICc = 28.43.

Again, the residuals of the three final models (with normal distribution in all cases) were not spatially structured according to Moran's I index.

### The Sicily case study

**Figure 2:**
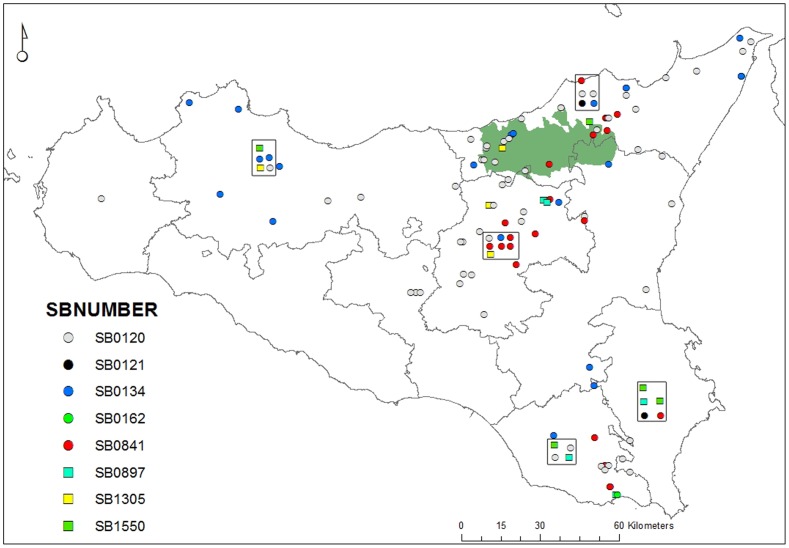
*Mycobacterium bovis* isolates from cattle by spoligotype pattern in Sicily. Map of Sicily. Solid grey lines indicate province boundaries. Dots represent individual *Mycobacterium bovis* isolates from cattle by spoligotype pattern. Dots in squares represent isolates with unknown geographical origin. Only the frequent patterns are represented. The area of the Nebrodi park, where free-ranging pigs contribute to *M. bovis* maintenance is shadowed. Cases are more frequent close to Nebrodi and close to Hyblaean Mountains and Ragusa (in the South) where there are high numbers of dairy cattle.

We identified 19 different *M. bovis* spoligotype patterns (Table 3) and one *M. caprae* pattern (SB0418) among 145 MTC isolates from Sicilian cattle in 2010 and 2011 (Table S1). Cattle farms were concentrated in the north west of Sicily, often coinciding with natural parks such as Nebrodi (Fig. 2). Table 4 compares the spoligotypes found in Sicily with those reported for the Italian mainland, where isolate sampling methods were similar. The three most common spoligotype patterns coincided between Sicily and mainland Italy. However, the frequency distribution of these three patterns was different (χ^2^ = 40.5, 2 d.f., p<0.001) due to a clearer dominance of the common patterns in Sicily (85% of isolates in only three patterns). On Sicily, SB0120 was present in all seven provinces with samples available. Necropsies on two fallow deer from one game park revealed TB-compatible lesions and allowed isolating *M. bovis*. The spoligotype pattern found was also SB0120. Two spoligotype patterns were new and consequently added to the database with the references SB2061 and SB2063. Hence, seven of 19 patterns (37%) found on Sicily had not been reported from continental Italy, representing 10 of 147 isolates (6.8%). Finally, no statistically significant spatial clusters of spoligotypes were detected in Sicily.

**Table 3:** *Mycobacterium bovis* spoligotype patterns identified in strains isolated from cattle from Sicily.

SB number	Binary code
SB0120	▪▪□▪▪▪▪▪□▪▪▪▪▪▪□▪▪▪▪▪▪▪▪▪▪▪▪▪▪▪▪▪▪▪▪▪▪□□□□□
SB0121	▪▪□▪▪▪▪▪□▪▪▪▪▪▪□▪▪▪▪□▪▪▪▪▪▪▪▪▪▪▪▪▪▪▪▪▪□□□□□
SB0134	▪▪□□□▪▪▪□▪▪▪▪▪▪□▪▪▪▪▪▪▪▪▪▪▪▪▪▪▪▪▪▪▪▪▪▪□□□□□
SB0162	□□□□□□□□□□□□□□□□□□□□□□□□▪▪▪▪▪▪▪▪▪▪▪▪▪▪□□□□□
SB0828	▪▪□▪▪▪▪▪□▪▪▪▪▪▪□▪▪▪▪▪▪▪▪▪▪▪▪▪▪▪▪▪□▪▪▪▪□□□□□
SB0841	▪▪□▪▪□□▪□▪▪▪▪▪▪□▪▪▪▪▪▪▪▪▪▪▪▪▪▪▪▪▪▪▪▪▪▪□□□□□
SB0850	▪▪□▪▪▪▪▪□▪▪▪▪▪▪□▪▪▪▪▪▪▪▪▪▪▪▪▪□□▪▪▪▪▪▪▪□□□□□
SB0897	▪▪□▪▪▪▪▪□▪▪□▪▪▪□▪▪▪▪▪▪▪▪▪▪▪▪▪▪▪▪▪▪▪▪▪▪□□□□□
SB0961	▪□□▪▪▪▪▪□▪▪▪▪▪▪□▪▪▪▪▪▪▪▪▪▪▪▪▪▪▪▪▪▪▪▪▪▪□□□□□
SB1305	▪□□□▪▪▪▪□▪▪▪▪▪▪□▪▪▪▪□▪▪▪▪▪□▪▪▪▪▪▪▪▪▪▪▪□□□□□
SB1335	▪▪□▪▪▪▪▪□▪▪▪▪▪▪□▪▪▪▪□▪▪▪▪▪▪▪▪□□▪▪▪▪▪▪▪□□□□□
SB1550	▪▪□▪▪▪▪□□▪▪▪▪▪▪□▪▪▪▪▪▪▪▪▪▪▪▪▪▪▪▪▪▪▪▪▪▪□□□□□
SB1567	▪▪□▪▪▪▪▪□▪▪▪▪▪▪□▪▪▪▪▪▪▪▪▪▪▪□□□□▪▪▪▪▪▪▪□□□□□
SB1569	▪▪□▪▪▪▪▪□▪▪▪▪▪▪□▪▪▪▪▪▪▪▪▪▪▪▪□□▪▪▪▪▪▪▪▪□□□□□
SB1945	▪▪□□□▪▪□□□□□□□▪□▪▪▪▪▪▪▪▪▪▪▪▪▪▪▪▪▪▪▪▪▪▪□□□□□
SB1946	▪▪□□□▪▪▪□▪▪▪▪▪▪□▪▪□▪▪▪▪▪▪▪▪▪▪▪▪▪▪▪▪▪▪▪□□□□□
SB1999	▪▪□▪▪□□▪□▪▪▪▪▪▪□▪▪▪▪▪▪▪▪▪▪▪▪▪▪□▪▪▪▪▪▪▪□□□□□
SB2061	▪▪□▪▪▪▪▪□▪▪▪▪▪▪□□▪▪▪▪▪▪▪▪▪▪▪▪▪▪▪▪□□▪▪▪□□□□□
SB2063	▪▪□▪▪▪▪□□▪▪▪▪▪□□□□▪▪▪▪▪▪▪▪▪▪▪▪▪▪▪▪▪▪▪▪□□□□□

The ▪ indicates spacer presence while □ indicates the absence of the spacer.

**Table 4:** *Mycobacterium bovis* spoligotype patterns identified from Sicily (this study) and mainland Italy (adapted from Boniotti et al. [63]).

Spoligotype pattern	Sicily (N = 147; 19 patterns)	Italian mainland (N = 747; 81 patterns)
SB0120	63 (43.2%)	408 (54.6%)
SB0134	25 (16.9%)	43 (5.7%)
SB0841	24 (16.2%)	36 (4.8%)
Other	35 (23.6%)	260 (34.8%)

Number of isolates and percentage are shown.

## Discussion

Island biogeography processes such as extinction or survival, and speciation or adaptation, are driven by factors such as contact with the mainland, island size and habitat–or host–diversity. By combining information available from the literature on old world islands with original information on Sicily, we were able to infer several aspects of MTC epidemiology and control in fragmented pathogen populations. Thus, this manuscript should be understood as an attempt of shortening distances between epidemiology and biogeography under the general aim of contributing to the study of animal health from an interdisciplinary perspective [47]–[48]. Insight gained in this study may also be extended to other islands and to “conceptual” islands, such as administrative units.

The fact that several small islands are *M. bovis* free could represent an example of the decreasing species survival as island size decreases [49]. We found that cattle bTB herd prevalence was positively correlated with island size but also with the commercial network intensity. From an epidemiological point of view, small islands import fewer animals and make movement restrictions easy, and smaller total host populations make disease eradication more likely than on the mainland. Bluetongue virus, for instance, was successfully controlled by sheep and cattle vaccination and movement restriction on the Balearic Islands [50], while bluetongue virus control in the European mainland has not yet been achieved [51]. Differences in the bTB control history might explain the variations observed among larger islands [27].

Another insight from our study is that bTB eradication in cattle will prove more difficult on islands with complex host communities. For example, TB persisted in feral pigs in Molokai island (Hawaii) after the removal of the cattle stock, which became infected again when reintroduced [52]. In this way, according to the theory of the ecosystem service of biodiversity–from which we expect that biodiversity regulates disease emergence and spread –, this scenario cannot be generalized for pathogens mainly circulating in wild reservoirs in which reducing biodiversity increases disease transmission [53]. In our sample, MTC strain diversity was positively related to the presence of wild hosts and the number of imported cattle, but not with island size. The relevance of wild hosts both for bTB prevalence and for MTC diversity in islands is coherent with findings from mainland populations [26]. At this respect, the case of badgers in Britain and Ireland, even when it likely represents an extreme in the role of wildlife on bTB epidemiology in islands, is a well-studied example [54]. Accordingly, the introduction of wildlife species to islands (e.g. wild boar in Britain, [55]) should be strictly regulated, obviously, from an ecological perspective [56], but also from a sanitary point of view, since epidemiological complexity of the systems increases and the success of disease control programs can be constrained [57].

The importance of animal movements in bTB epidemiology at large spatial scales is well known. For example, risk factor analyses carried out recently in Belgium emphasized the role of animal movements in the transmission of MTC infection [58]. According to our results, the effects of animal movements are relevant in the context of bTB epidemiology in islands as they increase the probability of infection entrance into a population [37]. As expected, the potential effect of insularity on MTC strain diversity was not quite evident in this study. This was likely because geographic isolation does not necessarily explain endoparasite frequency and diversity on livestock, since the probability for endoparasite entrance into an island, or any isolated population, depends more on the intensity of commercial networks than on geographical distance [27], [37]. From an epidemiological perspective, controlling trade movements is easier in islands (mainly in the smaller ones) making it feasible to substantially reduce this risk factor and therefore disease rates [59]. Furthermore, strain diversity may also be the consequence of human impact through test and slaughter, and that this could have contributed to the relatively low strain diversity in the British islands [27].

On Sicily, the larger concentration of cases in the northeast around the Nebrodi park (see Fig. 2), along with the recent description of the Sicilian black pig as a MTC reservoir [43], strongly suggest that the existence of multiple suitable MTC hosts is contributing to the local survival of the pathogen. This type of extensive cattle farming and coexistence of other potential wild and domestic hosts is similar to that described for Mediterranean habitats of the Iberian Peninsula [60]. On Sicily, SB0120 represented 74% of the Sicilian black pig isolates and SB0841 was also present in 16% of the *M. bovis* infected Sicilian black pigs [43]. Interestingly, strains SB0833 and SB2018, which have been recorded in Sicilian black pigs [43], were not detected in our Sicilian cattle strain sample. These results have implications for global bTB control: eradication will prove more difficult with increasing size of the island and its environmental complexity, mainly in terms of the diversity of suitable domestic and wild MTC hosts.

The Azores *M. bovis* outbreak in 2007 was characterized as a recent introduction with evolution of several strains from a common founder [30]. Similarly, new *M. bovis* types, such as SB2061 and SB2063 in this study and others found recently in Sicilian black pigs [43], also suggest recent evolution of MTC strains in islands. In this way, our results showed that 41% of the patterns isolated in Sicily had not been reported in mainland Italy. These facts, together with the positive association between MTC strain diversity and presence of wild hosts, would reinforce the hypothesis of higher rates of speciation on islands, a phenomenon likely to occur when organisms adapt to new–geographical and biological–environments [61].

Sicily is very close to the Italian mainland and cattle movements through the Messina strait are common. Thus, it is not surprising that Sicilian MTC isolates showed a link to isolates from mainland Italy (but they quantitatively differed; see below), with a few types in common only with French and African isolates and some unique strains [62]–[65]. The deletion of spacer 21 is a characteristic of the European 2 clonal complex of *M. bovis* (EU2). This complex is dominant in the Iberian Peninsula, where it represents almost ¾ of all isolates, but is rarely found on the Italian mainland (4%; [66]). On Sicily, strains lacking spacer 21 were observed at the same rate as in mainland Italy. One of the Sicilian strains (SB1945) lacked spacer 11 and thus may belong to the European 1 clonal complex of *M. bovis*, dominant on the British Islands [27]. These strains were detected in less than 1% of strains from mainland Italy [63], [67]. We also found no strains with the characteristic deletions of the African 1 and African 2 clonal complexes. The low frequency of *M. caprae* strains in Sicily (one isolate, 1%) was in agreement with the north-south declining gradient in *M. caprae* prevalence observed in mainland Italy [63]. From a bTB control perspective, this reflects the fact that re-colonization of islands close to (infected) mainland regions is more likely than of more distant islands or archipelagos. This suggests that bTB control schemes should be designed based on large, uniformly controlled geographical units rather than leaving pockets of infection, progressively extending from one to the other extreme of the total infected region. However on Sicily, bTB epidemiology may also be affected by other factors. For instance, a European Comission audit on the bTB eradication scheme stated that “the autonomous region of Sicily has implemented their programme to a much lesser extent, largely due to fundamental difficulties in applying basic legislation on animal identification and movement controls foreseen in EU legislation” [ec.europa.eu/food/fvo/act_getPDF.cfm?PDF_ID...]. This could also explain the lack of spatial structuring of MTC strains observed in this study.

Our results showed differences between Sicily and mainland Italy in relation to the frequency of MTC strains isolated. While on Sicily only three MTC strains constituted 85% of the isolates, in mainland Italy these strains represented 65% of the isolates (Table 4; [63]). This could be related to the global processes in islands by which these ecosystems tend to be dominated by a reduced number of–generalist–species. A founder effect, caused by the predominant of a limited number of strains historically introduced, can be more evident in Islands than in continental lands [30]. These evidences should be taken into account when designing disease control programs to be applied in areas of different size and biogeographical characteristics.

Summarizing, this study showed that island size, host diversity and human factors such as the intensity of commercial networks and the implementation of bTB control schemes affect bTB prevalence and MTC strain diversity on islands, providing new insights for TB control and opening avenues for further research.

## Supporting Information

Table S1 **Raw information about the** ***Mycobacterium bovis*** **isolates from cattle in Sicily; year of isolation and location by spoligotype pattern are reported.**
(DOCX)

## References

[1] MacArthur RH, Wilson EO (1967) The theory of island biogeography. Princeton University Press, Princeton.

[2] Whittaker RJ, Fernández-Palacios JM (2007) Island biogeography: ecology, evolution, and conservation, 2nd edn. Oxford University Press, Oxford.

[3] Losos JB, Ricklefs RE (2010) The theory of island biogeography revisited. Princeton University Press, Woodstock.

[4] Baldi A, McCollin D (2003) Island ecology and contingent theory: the role of spatial scale and taxonomic bias. Glob Ecol Biogeogr 12: 1–3.

[5] Gillespie RG, Roderick GK (2002) Arthropods on islands: Colonization, speciation, and conservation. Annu Rev Entomol 47: 595–632. 11729086 10.1146/annurev.ento.47.091201.145244

[6] Lloret F, Medail F, Brundu G, Camarda I, Moragues E, et al (2005) Species attributes and invasion success by alien plants on Mediterranean islands. J Ecol 93: 512–520.

[7] Fernandez-Palacios JM, de Nascimento L, Otto R, Delgado JD, Garcia-del-Rey E, et al (2011) A reconstruction of Palaeo-Macaronesia, with particular reference to the long-term biogeography of the Atlantic island laurel forests. J Biogeogr 38: 226–246.

[8] Delahay RJ, Smith GC, Hutchings R (2009) Management of disease in wild mammals. Springer, Tokio.

[9] Apanius V, Yorinks N, Bermingham E, Ricklefs RE (2000) Island and taxon effects in parasitism and resistence of lesser antillean birds. Ecology 81: 1959–1969.

[10] Fromont E, Morvilliers L, Artois M, Pontier D (2001) Parasite richness and abundance in insular and mainland feral cats : insularity or density? Parasitology 123: 143–151. 11510679 10.1017/s0031182001008277

[11] Goüy de Bellocq J, Morand S, Feliu C (2002) Patterns of parasite species richness of Western Palaeartic: micro-mammals: island effects. Ecography 25: 173–183.

[12] Goüy de Bellocq J, Sarà M, Casanova JC, Feliu C, Morand S (2003) A comparison of the structure of helminth communities in the woodmouse, *Apodemus sylvaticus*, on islands of the western Mediterranean and continental Europe. Parasitol Res 90: 64–70. 12743806 10.1007/s00436-002-0806-1

[13] Ishtiaq F, Clegg SM, Phillimore AB, Black RA, Owens IPF, et al (2010) Biogeographical patterns of blood parasite lineage diversity in avian hosts from southern Melanesian islands. J Biogeogr 37: 120–132.

[14] Kuris AM, Blaunstein AR, Alio JJ (1980) Hosts as islands. Am Nat 116: 570–586.

[15] Poulin R (2004) Macroecological patterns of species richness in parasite assemblages. Basic Appli Ecol 5: 423–434.

[16] Reperant LA (2010) Applying the Theory of Island Biogeography to Emerging Pathogens: Toward Predicting the Sources of Future Emerging Zoonotic and Vector-Borne Diseases. Vector-Borne Zoonot 10: 105–110. 10.1089/vbz.2008.0208 19589061

[17] Bowman DD (2006) Successful and currently ongoing parasite eradication programs. Vet Parasitol 139: 293–307. 16730411 10.1016/j.vetpar.2006.04.020

[18] Smith NH, Gordon SV, Rua-Domenech R, Clifton-Hadley RS, Hewinson RG (2006) Bottlenecks and broomsticks: the molecular evolution of *Mycobacterium bovis* . Nat Rev Microbiol 4: 670–681. 16912712 10.1038/nrmicro1472

[19] Rodriguez-Campos S, Schürch AC, Dale J, Lohan AJ, Cunha MV, et al (2012) European 2–A clonal complex of *Mycobacterium bovis* dominant in the Iberian Peninsula. Infect Genet Evol 12: 866–872. 21945286 10.1016/j.meegid.2011.09.004

[20] Brosch R, Gordon SV, Marmiesse M, Brodin P, Buchrieser C, et al (2002) A new evolutionary scenario for the Mycobacterium tuberculosis complex. P Natl Acad Sci USA 99: 3684–9. 10.1073/pnas.052548299 PMC122584 11891304

[21] O'Reilly LM, Daborn CJ (1995) The epidemiology of *Mycobacterium bovis* infections in animals and man: a review. Tuber Lung Dis 76: 1–46. 10.1016/0962-8479(95)90591-x 7579326

[22] Zinsstag J, Schelling E, Roth F, Kazwala R (2006) Economics of bovine tuberculosis. In Thoen CO, Steele JH, Gilsdorf MJ (eds) *Mycobacterium bovis* infection in animals and human, Wiley-Blackwell, New York, 68–83.

[23] Durr PA, Hewinson RG, Clifton-Hadley RS (2000) Molecular epidemiology of bovine tuberculosis I *Mycobacterium bovis* typing. Rev Sci Tech Off Int Epiz 19: 675–688. 10.20506/rst.19.3.1241 11107611

[24] Gortazar C, Torres MJ, Acevedo P, Aznar J, Negro JJ, et al (2011) Fine-tuning the space, time, and host distribution of mycobacteria in wildlife. BMC Microbiol 11: 27. 21288321 10.1186/1471-2180-11-27 PMC3040691

[25] Gortazar C, Vicente J, Samper S, Garrido J, Fernandez-De-Mera IG, et al (2005) Molecular characterization of *Mycobacterium tuberculosis* complex isolates from wild ungulates in South-Central Spain. Vet Res 36: 43–52. 15610722 10.1051/vetres:2004051

[26] Gortazar C, Torres MJ, Vicente J, Acevedo P, Reglero M, et al (2008) Bovine tuberculosis in Doñana biosphere reserve: the role of wild ungulates as disease reservoirs in the last Iberian lynx strongholds. PLoS ONE 3: e2776. 18648665 10.1371/journal.pone.0002776 PMC2464716

[27] Smith NH, Berg S, Dale J, Allen A, Rodriguez S, et al (2011) European 1: a globally important clonal complex of *Mycobacterium bovis* . Infect Genet Evol 11: 1340–51. 21571099 10.1016/j.meegid.2011.04.027

[28] Caminero JA, Pena MJ, Campos-Herrero MI, Rodriguez JC, Garcia I, et al (2001) Epidemiological evidence of the spread of a *Mycobacterium tuberculosis* strain of the Beijing genotype on Gran Canaria Island. Am J Respir Crit Care Med 164: 1165–1170. 11673204 10.1164/ajrccm.164.7.2101031

[29] Millet J, Miyagi-Shiohira C, Yamane N, Sola C, Rastogi N (2007) Assessment of mycobacterial interspersed repetitive unit-QUB markers to further discriminate the Beijing genotype in a population-based study of the genetic diversity of Mycobacterium tuberculosis clinical isolates from Okinawa, Ryukyu Islands, Japan. J Clin Microbiol 45: 3606–3615. 17898160 10.1128/JCM.00348-07 PMC2168487

[30] Matos F, Amado A, Botelho A (2010) Molecular typing of *Mycobacterium bovis* isolated in the first outbreak of bovine tuberculosis in the Azores Islands: a case report. Vet Med-Czech 55: 133–136.

[31] Richomme C, Boschiroli ML, Hars J, Casabianca F, Ducrot C (2010) Bovine Tuberculosis in Livestock and Wild Boar on the Mediterranean Island, Corsica. J Wildlife Dis 46: 627–631. 10.7589/0090-3558-46.2.627 20688663

[32] Walther BA, Cotgreave P, Price RD, Gregory RD, Clayton DH (1995) Sampling effort and parasite species richness. Parasitol Today 11: 306–310. 15275331 10.1016/0169-4758(95)80047-6

[33] Margalef R (1958) Information theory in ecology. General Syst 3: 36–71.

[34] Magurran AE (2004) Measuring Biological Diversity. Blackwell Publishing, London.

[35] Arneberg P, Skorping A, Grenfell B, Read AF (1998) Host densities as determinants of abundance in parasite communities. P Roy Soc B-Biol Sci 265: 1283–1289.

[36] Gortazar C, Delahay RJ, McDonald RA, Boadella M, Wilson GJ, et al (2012) The status of tuberculosis in European wild mammals. Mammal Rev 42: 193–206.

[37] Gilbert M, Mitchell A, Bourn D, Mawdsley J, Clifton –Hadley R, et al (2005) Cattle movements and bovine tuberculosis in Great Britain. Nature 435(7041): 491–6. 15917808 10.1038/nature03548

[38] McCullagh P, Nelder JA (1989) Generalized linear models. Chapman & Hall, London.

[39] Akaike H (1974) A new look at the statistical model identification. IEEE Trans Autom Cont 19: 716 –723.

[40] Diniz-Filho JAF, Bini LM, Hawkins BA (2003) Spatial autocorrelation and red herrings in geographical ecology. Glob Ecol Biogeogr 12: 53–64.

[41] Benjamin S (2007) Sicily: Three Thousand Years of Human History. Trade paperback, Steerforth Press, Hanover.

[42] Caracappa S (1999) Livestock production and animal health in Sicily, Italy. Parassitologia 41: 17–23. 11071536

[43] Di Marco V, Mazzone P, Capucchio MT, Boniotti MB, Aronica V, et al (2012) Epidemiological Significance of the Domestic Black Pig (*Sus scrofa*) in Maintenance of Bovine Tuberculosis in Sicily. J Clin Microbiol 50: 1209–1218. 22322347 10.1128/JCM.06544-11 PMC3318573

[44] Masseti M, Mertzanidou D (2008) *Dama dama* In: IUCN 2011. IUCN Red List of Threatened Species. Version 2011.2. Available: www.iucnredlist.org. Accessed 2012 February 23.

[45] Kamerbeek J, Schouls L, Kolk A, van Agterveld M, van Soolingen D, et al (1997) Simultaneous detection and strain differentiation of *Mycobacterium tuberculosis* for diagnosis and epidemiology. J Clin Microbiol 35: 907–914. 9157152 10.1128/jcm.35.4.907-914.1997 PMC229700

[46] Smith NH, Upton P (2012) Naming spoligotype patterns for the RD9-deleted lineage of the *Mycobacterium tuberculosis* complex; www.Mbovis.org. Infect Genet Evol. 12: 873–876. 10.1016/j.meegid.2011.08.002 21855653

[47] Scheiner SM (2010) The intersection of the sciences of biogeography and infectious diseases ecology. EcoHealth 6: 483–488. 10.1007/s10393-010-0298-x 20419511

[48] Acevedo P, Real R (2012) Favourability: concept, distinctive characteristics and potential usefulness. Naturwissenschaften 99: 515–522. 22660474 10.1007/s00114-012-0926-0

[49] Wilcox BA, Murphy DD (1985) Conservation Strategy: The Effects of Fragmentation on Extinction. Am Nat 125: 879–887.

[50] Gómez-Tejedor C (2004) Brief overview of the bluetongue situation in Mediterranean Europe, 1998–2004. Vet Ital 40: 57–60. 20419636

[51] Falconi C, López-Olvera J, Gortazar C (2011) BTV infection in wild ruminants, with emphasis on red deer: a review. Vet Microbiol 151: 209–219. 21411246 10.1016/j.vetmic.2011.02.011

[52] Meyer RM (1998) Bovine TB on Molokai Island Results of a Wildlife Disease Survey to Date. Proceedings of the 102nd Annual Meeting of the United States Animal Health Association, Minneapolis, Minnesota.

[53] Keesing F, Belden LK, Daszak P, Dobson A, Harvell CD, et al (2010) Impacts of biodiversity on the emergence and transmission of infectious diseases. Nature 468: 647–652. 21124449 10.1038/nature09575 PMC7094913

[54] Krebs JR, Anderson R, Clutton-Brock T, Morrison I, Young D, et al. (1997) Bovine tuberculosis in cattle and badgers. Her Majesty's Stationary Office, London, UK.

[55] Wilson CJ (2003) Distribution and status of feral wild boar *Sus scrofa* in Dorset, southern England. Mammal Rev 33: 302–307.

[56] Courchamp F, Chapuis JL, Pascal M (2003) Mammal invaders on islands: impact, control and control impact. Biol Rev 78: 347–383. 14558589 10.1017/s1464793102006061

[57] Hartley M (2010) Qualitative risk assessment of the role of the feral wild boar (*Sus scrofa*) in the likelihood of incursion and the impacts on effective disease control of selected exotic diseases in England. Eur J Wildlife Res 56: 401–410.

[58] Humblet MF, Gilbert M, Govaerts M, Fauville-Dufaux M, Walravens K, et al (2010) New assessment of bovine tuberculosis risk factors in Belgium based on nationwide molecular epidemiology. J Clin Microbiol 48: 2802–2808. 20573869 10.1128/JCM.00293-10 PMC2916604

[59] Gibbens JC, Sharpe CE, Wilesmith JW, Mansley LM, Michalopoulou E, et al (2001) Descriptive epidemiology of the 2001 foot and mouth disease epidemic in Great Britain: The first five months. Vet Rec 149: 729–743. 11808655

[60] Gortazar C, Vicente J, Boadella M, Ballesteros C, Galindo RC, et al (2011) Progress in the control of bovine tuberculosis in Spanish wildlife. Vet Microbiol 151: 170–178. 21440387 10.1016/j.vetmic.2011.02.041

[61] Vellend M (2003) Island biogeography of genes and species. Am Nat 162: 358–365. 12970843 10.1086/377189

[62] Haddad N, Ostyn A, Karoui C, Masselot M, Thorel MF, et al (2001) Spoligotype diversity of *Mycobacterium bovis* strains isolated in France from 1979 to 2000. J Clin Microbiol 39: 3623–3632. 11574583 10.1128/JCM.39.10.3623-3632.2001 PMC88399

[63] Boniotti MB, Goria M, Loda D, Garrone A, Benedetto A, et al (2009) Molecular typing of *Mycobacterium bovis* strains isolated in Italy from 2000 to 2006 and evaluation of variable-number tandem repeats for geographically optimized genotyping. J Clin Microbiol 47: 636–644. 19144792 10.1128/JCM.01192-08 PMC2650904

[64] Munyeme M, Muma JB, Samuui KL, Skjerve E, Nambota AM, et al (2009) Prevalence of bovine tuberculosis and animal level risk factors for indigenous cattle under different grazing strategies in the livestock/wildlife interface areas of Zambia. Trop Anim Health Pro 41: 345–352. 10.1007/s11250-008-9195-5 18536998

[65] Sahraoui N, Müller B, Guetarni D, Boulahbal F, Yala D, et al (2009) Molecular characterization of *Mycobacterium bovis* strains isolated from cattle slaughtered at two abattoirs in Algeria. BMC Vet Res 5: 4. 19173726 10.1186/1746-6148-5-4 PMC2640374

[66] Rodriguez-Campos S, Gonzalez S, de Juan L, Romero B, Bezos J, et al (2012) A database for animal tuberculosis (mycoDB.es) within the context of the Spanish national programme for eradication of bovine tuberculosis. Infect Genet Evol 12: 877–882. 22027158 10.1016/j.meegid.2011.10.008

[67] Smith NH (2012) The global distribution and phylogeography of *Mycobacterium bovis* clonal complexes. Infect Genet Evol 12: 857–65. 21945588 10.1016/j.meegid.2011.09.007

[68] Skuce RA, Mallon TR, McCormick CM, McBride SH, Clarke G, et al (2010) *Mycobacterium bovis* genotypes in Northern Ireland: herd-level surveillance (2003 to 2008). Vet Rec 167: 684–689. 21257483 10.1136/vr.c5108

[69] ANSES (2010) French Agency for Food and Environmental and Occupational Health & Safety. Internal report.

[70] Rodríguez S, Romero B, Bezos J, de Juan L, Alvarez J, et al (2010) High spoligotype diversity within a *Mycobacterium bovis* population: clues to understanding the demography of the pathogen in Europe. Vet Microbiol 141: 89–95. 19720476 10.1016/j.vetmic.2009.08.007

[71] Duarte EL, Domingos M, Amado A, Botelho A (2008) Spoligotype diversity of *Mycobacterium bovis* and *Mycobacterium caprae* animal isolates. Vet Microbiol 130: 415–421. 18417301 10.1016/j.vetmic.2008.02.012

