# Supplementary material for: Tuberculosis Epidemiology in Islands: Insularity, Hosts and Trade
Source: PLoS One. 2013 Jul 29;8(7):e71074. doi: 10.1371/journal.pone.0071074 (PMC3726611; doi:10.1371/journal.pone.0071074)
Supplement: Table S1 — Raw information about the Mycobacterium bovis isolates from cattle in Sicily; year of isolation and location by spoligotype pattern are reported. (DOCX) [file pone.0071074.s001.docx]

**Table S1**. Raw information about the *Mycobacterium bovis* isolates from cattle in Sicily; year of isolation and location by spoligotype pattern are reported.

| **Spoligotype pattern**  **(# isolates when >1)** | **Year** | **Province** – **municipality** |
| --- | --- | --- |
| SB0120 (3) | 2011 | Caltanissetta – Caltanissetta |
| SB0120 | 2011 | Catania – Castel di Luica |
| SB0120 | 2010 | Catania – Catania |
| SB0120 | 2011 | Catania – Giarre |
| SB0120 |  | Enna |
| SB0120 (2) | 2011 | Enna – Calascibetta |
| SB0120 | 2010 | Enna – Centuripe |
| SB0120 | 2010 | Enna – Cerami |
| SB0120 | 2011 | Enna – Cerami |
| SB0120 (2) | 2010 | Enna – Enna |
| SB0120 | 2011 | Enna – Enna |
| SB0120 | 2010 | Enna – Leonforte |
| SB0120 | 2011 | Enna – Nicosia |
| SB0120 | 2010 | Enna – Piazza Armerina |
| SB0120 |  | Messina |
| SB0120 | 2011 | Messina – Barcellona Pozzo di Gotto |
| SB0120 | 2010 | Messina – Capizzi |
| SB0120 (3) | 2010 | Messina – Caronia |
| SB0120 | 2011 | Messina – Caronia |
| SB0120 | 2010 | Messina – Castelferrato |
| SB0120 | 2010 | Messina – Castroreale |
| SB0120 | 2011 | Messina – Domenica |
| SB0120 | 2010 | Messina – Messina |
| SB0120 (2) | 2011 | Messina – Messina |
| SB0120 (3) | 2010 | Messina – Mistretta |
| SB0120 (2) | 2011 | Messina – Mistretta |
| SB0120 | 2011 | Messina – Moio Alcantara |
| SB0120 | 2010 | Messina – Montalbano Elicona |
| SB0120 | 2011 | Messina – Motta d´Affermo |
| SB0120 | 2010 | Messina – Pace del Mela |
| SB0120 | 2011 | Messina – Raccuja |
| SB0120 (4) | 2010 | Messina – San Marco D´Alunzio |
| SB0120 | 2010 | Messina – Tortorici |
| SB0120 |  | Palermo |
| SB0120 | 2011 | Palermo – Bisacquino |
| SB0120 | 2010 | Palermo – Caccamo |
| SB0120 | 2010 | Palermo – Geraci Siculo |
| SB0120 | 2011 | Palermo – Sclafani Bagni |
| SB0120 | 2010 | Ragusa |
| SB0120 | 2010 | Ragusa – Ispica |
| SB0120 (3) | 2011 | Ragusa – Modica |
| SB0120 | 2011 | Ragusa – Ragusa |
| SB0120 | 2011 | Siracusa – Carlentini |
| SB0120 (2) | 2010 | Siracusa – Noto |
| SB0120 (2) | 2010 | Siracusa – Rosolini |
| SB0120 |  | Trapani |
| SB0121 |  | Messina |
| SB0121 |  | Siracusa |
| SB0134 | 2010 | Catania – Randazzo |
| SB0134 | 2010 | Catania – Randazzo |
| SB0134 |  | Enna |
| SB0134 | 2011 | Enna – Castelferrato |
| SB0134 | 2010 | Enna – Troina |
| SB0134 |  | Messina |
| SB0134 (2) | 2010 | Messina – Caronia |
| SB0134 (5) | 2010 | Messina – Messina |
| SB0134 | 2011 | Messina – Mistretta |
| SB0134 (2) | 2010 | Messina – Patti |
| SB0134 (2) |  | Palermo |
| SB0134 | 2011 | Palermo – Cinisi |
| SB0134 | 2011 | Palermo – Corleone |
| SB0134 | 2011 | Palermo – Palermo |
| SB0134 | 2011 | Palermo – Prizzi |
| SB0134 | 2011 | Palermo – Villafrati |
| SB0134 | 2011 | Ragusa – Comiso |
| SB0134 | 2010 | Ragusa – Giarratana |
| SB0162 | 2010 | Catania – Biancavilla |
| SB0162 | 2011 | Ragusa – Pozzallo |
| SB0418 |  | Ragusa |
| SB0828 | 2010 | Enna – Nicosia |
| SB0841 | 2011 | Catania – Biancavilla |
| SB0841 (4) |  | Enna |
| SB0841 | 2010 | Enna – Agira |
| SB0841 | 2011 | Enna – Assoro |
| SB0841 | 2010 | Enna – Centuripe |
| SB0841 | 2011 | Enna – Centuripe |
| SB0841 | 2011 | Enna – Nissoria |
| SB0841 | 2011 | Enna – Troina |
| SB0841 |  | Messina |
| SB0841 | 2010 | Messina – Cesaro |
| SB0841 | 2011 | Messina – Domenica |
| SB0841 | 2010 | Messina – Floresta |
| SB0841 (2) | 2011 | Messina – Raccuja |
| SB0841 | 2011 | Messina – San Piero Patti |
| SB0841 | 2010 | Messina – Tortorici |
| SB0841 (4) | 2010 | Ragusa – Modica |
| SB0841 | 2010 | Ragusa – Ragusa |
| SB0841 |  | Siracusa |
| SB0850 |  | Palermo |
| SB0897 (3) | 2011 | Enna – Troina |
| SB0897 |  | Ragusa |
| SB0897 | 2011 | Ragusa – Pozzallo |
| SB0897 |  | Siracusa |
| SB0961 | 2010 | Messina – Valdemone |
| SB1305 |  | Enna |
| SB1305 | 2011 | Enna – Nicosia |
| SB1305 | 2010 | Messina – Caronia |
| SB1305 |  | Palermo |
| SB1335 | 2011 | Ragusa – Camerina |
| SB1550 | 2011 | Messina – Cesaro |
| SB1550 | 2010 | Messina – Tortorici |
| SB1550 |  | Palermo |
| SB1550 | 2010 | Ragusa |
| SB1550 (2) |  | Siracusa |
| SB1567 | 2010 | Enna – Cerami |
| SB1567 | 2011 | Messina – Tripi |
| SB1569 | 2011 | Agriento |
| SB1945 |  | Catania |
| SB1946 |  | Catania |
| SB1999 | 2010 | Messina – Tortorici |
| SB2061 | 2011 | Enna – Troina |
| SB2061 | 2010 | Messina – Basico |
| SB2063 | 2010 | Siracusa – Lentini |
